# Online Reviews of Health Care Facilities

**DOI:** 10.1001/jamanetworkopen.2025.24505

**Published:** 2025-08-01

**Authors:** Neil K.R. Sehgal, Sharath Chandra Guntuku, Lauren Southwick, Raina M. Merchant, Anish K. Agarwal

**Affiliations:** 1 Computer and Information Science Department, University of Pennsylvania, Philadelphia; 2 Leonard Davis Institute of Health Economics, University of Pennsylvania, Philadelphia; 3 Penn Medicine Center for Health Care Transformation and Innovation, University of Pennsylvania, Philadelphia; 4 Department of Emergency Medicine, University of Pennsylvania Perelman School of Medicine, Philadelphia

## Abstract

**Importance:**

Understanding patient experience is crucial for improving health care delivery. However, language patterns and themes correlated with negative or positive ratings are not well known.

**Objective:**

To examine online reviews of US health care facilities, identifying language patterns and themes associated with negative or positive ratings.

**Design, Setting, and Participants:**

For this cross-sectional study, all reviews of US health care facilities offering essential health benefits, as defined by the Affordable Care Act, posted on 1 online platform (Yelp.com) under “Health & Medical” from January 1, 2017, to December 31, 2023, were obtained. Reviews are posted voluntarily with ratings (1 star = lowest, 5 stars = highest) and open-ended review narratives regarding patients’ care experiences.

**Main Outcomes and Measures:**

The primary outcome was the correlation between n-grams (1- to 3-word sequences) and review ratings (negative: 1 or 2 stars; positive: 4 or 5 stars). Secondary measures included linguistic analysis and topic modeling based on standard machine-learning algorithms. Machine-learning and natural-language processing, including n-gram correlation, linguistic feature analysis, and topic modeling, were applied to determine correlations with review star ratings.

**Results:**

A total of 1 099 901 online reviews from 138 605 facilities were identified over the 7-year study period. Among these, nearly one-half (46.3%) were negative and one-half (50.1%) were positive, with a median (IQR) rating of 4 (1-5) stars. The word “not” was most correlated with negative ratings (*r* = 0.31; 95% CI, 0.31–0.32), whereas “and” was most correlated with positive ratings (*r* = 0.35; 95% CI, 0.35–0.36). Among 200 topics, the strongest negative correlations involved payment issues (*r* = 0.25; 95% CI, 0.25–0.25) and poor treatment (*r* = 0.24; 95% CI, 0.23–0.24); the strongest positive correlations involved kindness (*r* = 0.32; 95% CI, 0.32–0.32) and anxiety relief (*r* = 0.32; 95% CI, 0.32–0.32).

**Conclusions and Relevance:**

In this cross-sectional analysis, negative patient experiences frequently centered on quality of communication and administrative issues. Negative feedback centered on unmet expectations, whereas positive reviews emphasized supportive staff interactions. Incorporating real-time online-review data into existing quality-improvement frameworks—such as patient experience dashboards or service recovery protocols—could help clinicians, administrators, and policymakers identify emerging concerns, monitor patient sentiment, and tailor interventions that enhance patient-centered care across diverse health care settings.

## Key Points

**Question:**

What words, linguistic categories, and themes correlate with highly positive or negative ratings in online reviews of health care?

**Findings:**

In this cross-sectional study of 1 099 901 online reviews posted from 2017 through 2023, 46.3% of US health care facilities were rated 1 to 2 stars and 50.1% were rated 4 to 5 stars. The word “not” and topics related to administrative barriers were significantly associated with negative ratings, whereas the conjunction “and” and words related to support staff interactions were associated with positive ratings.

**Meaning:**

Findings of this study suggest that assessing online-review language in real time could help clinicians and administrators identify possible emerging communication or access problems and target patient-centered quality-improvement interventions.

## Teaser

This cross-sectional study analyzes online reviews of patient experiences in US health care facilities, identifying words, linguistic categories, and themes correlated with highly positive or negative ratings.

## Introduction

Patient experience is a central component of providing high-quality health care, informing improvements in clinical care and organizational practices. Standardized surveys—such as those offered by Press Ganey^1^ or the Hospital Consumer Assessment of Healthcare Providers and Systems (HCAHPS)^2^—have helped follow patient experience scores at scale. These instruments provide robust, comparable metrics, yet their results are often difficult for patients or families to find, and their structured formats may constrain patient narratives—missing components important to patients and their families.

Many patients and their families now turn to public online review platforms^3^ to share health care experiences in their own words.^4^ These reviews create large datasets of patient-centered, unfiltered, and organic content, offering insights into communication patterns, service quality, and access challenges. Growing evidence suggests that online reviews can provide related and also different, nuanced elements of patient care compared with traditional surveys, including issues related to customer service, administrative efficiency, and staff interactions.^4,5,6,7,8,9,10^ For example, the HCAHPS survey does not include items on compassion of staff, yet prior work finds that many reviews of hospitals deal with experiences of (or a lack of) compassion.^4^ Unlike formal surveys, which rely on fixed-response questions and may constrain the expression of patient sentiment, online narratives can capture emotional tone (eg, frustration, gratitude), relational dynamics (eg, feelings of trust or being dismissed), and specific interactions often underrepresented in formal instruments—such as front-desk service, waiting room conditions, or communication between visits. These themes may emerge spontaneously in patient narratives, offering early signals of systemic issues or strengths that structured surveys may overlook.

Despite the value of online reviews, key gaps remain in our understanding of patient experiences and sentiment, and how they vary across different health care facility types and over time. While previous studies have shown that word choice on social media and rating platforms can reveal drivers of dissatisfaction or satisfaction, most analyses rely on data from earlier periods or focus on a single health care setting, such as urgent care centers.^7,11,12^

To address these gaps, this study analyzed reviews on an online platform (Yelp) of 20 different health care facility types from 2017 through 2023 using machine-learning and natural-language processing. Specifically, we used n-gram correlations, psycholinguistic feature analysis, and topic modeling to help identify the words, linguistic categories, and thematic topics most strongly correlated with highly positive or negative ratings.

Notably, this study period spans the COVID-19 pandemic, a time of unprecedented strain on health systems and substantial shifts in patient expectations, access, and care delivery. From 2020 to 2022, many facilities faced staffing shortages, operational disruptions, and evolving safety protocols—all of which may have shaped how patients experienced and evaluated their care. Online reviews written during this period may reflect not only individual encounters but also broader systemic challenges unique to the pandemic era.

By evaluating the breadth of current online patient narratives, this study aims to provide actionable insights for clinicians, administrators, and policymakers seeking to refine the patient experience in the post-pandemic era.

## Methods

We performed a retrospective analysis of US health care facility online reviews from January 1, 2017, to December 31, 2023. This platform is one of the most popular online-review forums, with more than 244 million users and approximately 73 million unique visitors daily.^13^ The Strengthening the Reporting of Observational Studies in Epidemiology (STROBE) reporting guideline was followed. The study was considered exempt under University of Pennsylvania Institutional Review Board guidelines because it did not meet the definition of human participant research.

An academic research dataset supplied directly by the online platform contained reviews from all US facilities tagged as health and medical, as outlined by the platform’s developer documentation. We filtered the dataset to focus specifically on facility subcategories aligned with essential health benefits, services mandated by the Affordable Care Act to be covered by health insurance plans (eMethods in Supplement 1).^10^ Each record contained the narrative text review, date of posting, and star rating (1 star [lowest rating] through 5 stars [highest rating]), facility name, facility category and subcategory, facility zip code, and review author’s name. Zip codes were mapped to zip code tabulation areas and joined with rural–urban commuting area codes to classify facilities as urban or rural.^14,15^

Reviews were tokenized into words, punctuation, and emoticons with the Happier Fun Tokenizer implemented in the Differential Language Analysis ToolKit (DLATK; version 1.3.0).^16^ We did not apply stemming, lemmatization, or language filtering. To enhance generalizability, we excluded words that appeared in less than 1% of all reviews and excluded the 50 most frequent words. This approach reduces the role of individual reviews in topic modeling and filters out overly common words (stop words) that offer little semantic value.

### Statistical Analysis

Using DLATK, we computed n-gram (1- to 3-word sequences) frequencies and their correlation with 1- or 2-star (negative) and 4- or 5-star (positive) reviews. Prior work has demonstrated that health care reviews in this online platform follow a bimodal distribution, with the vast majority classified as 1 or 5.^10^ DLATK uses an open-vocabulary approach, utilizing word frequency and Pearson correlation to identify correlations between specific words and review scores. We used linear regression where the outcome is the review scores (positive/negative) and the independent variable is the language feature.

To better understand correlations between negative and positive reviews with basic emotional and cognitive dimensions, we used linguistic features from the 2022 Linguistic Inquiry and Word Count (LIWC-22).^17^ Positive correlations represent greater correlations with positive reviews and negative correlations represent greater correlations with negative reviews.

Latent Dirichlet allocation (LDA), a probabilistic topic modeling technique, was applied to identify topics, clusters of co-occurring words, within the online platform’s narrative reviews. We selected 200 topics based on prior work using a similarly sized corpus of reviews on the online platform, where this number yielded a good balance of topic uniqueness and coherence.^10^ After generating 200 topics via the DLATK mallet interface, we examined the most highly correlated with topics with positive and negative reviews, providing insights into the recurring themes associated with patient sentiment. Because LDA assigns each review a distribution over topics rather than a single label, we reported topic prevalence—the average proportion of each topic across the dataset relative to other topics—as a measure of importance. To enhance interpretability, a clinician co-author (A.K.A.) examined the top words in each topic and assigned descriptive labels. This process has been used before in multiple analyses of online-review content.^4,10,11,12,18^ We used Python version 3.10 (Python Software Foundation) for all analyses.

## Results

Between January 1, 2017, and December 31, 2023, a total of 1 099 901 reviews were collected that were posted across 138 605 US health care facilities. The mean (SD) rating was 3.1 (1.92) stars (median [IQR], 4 [1-5] stars), with 46.3% of reviews rated as 1 or 2 stars (negative) and 50.1% rated as 4 or 5 stars (positive). The mean (SD) positive review length was 520.8 (468.98) characters (median [IQR], 387 [229-647] characters) and the mean (SD) negative review length was 785.8 (712.87) characters (median [IQR], 575 [319-992] characters). Facilities with the highest mean (SD) ratings included lactation services (4.65 [1.01]) and prenatal services (4.29 [1.46]). Facilities with the lowest mean (SD) ratings included hospitals (2.44 [1.82]) and pharmacies (2.55 [1.83]) (eTable in Supplement 1). Most of the reviews (97.1%) came from urban facilities.

**Figure 1.**
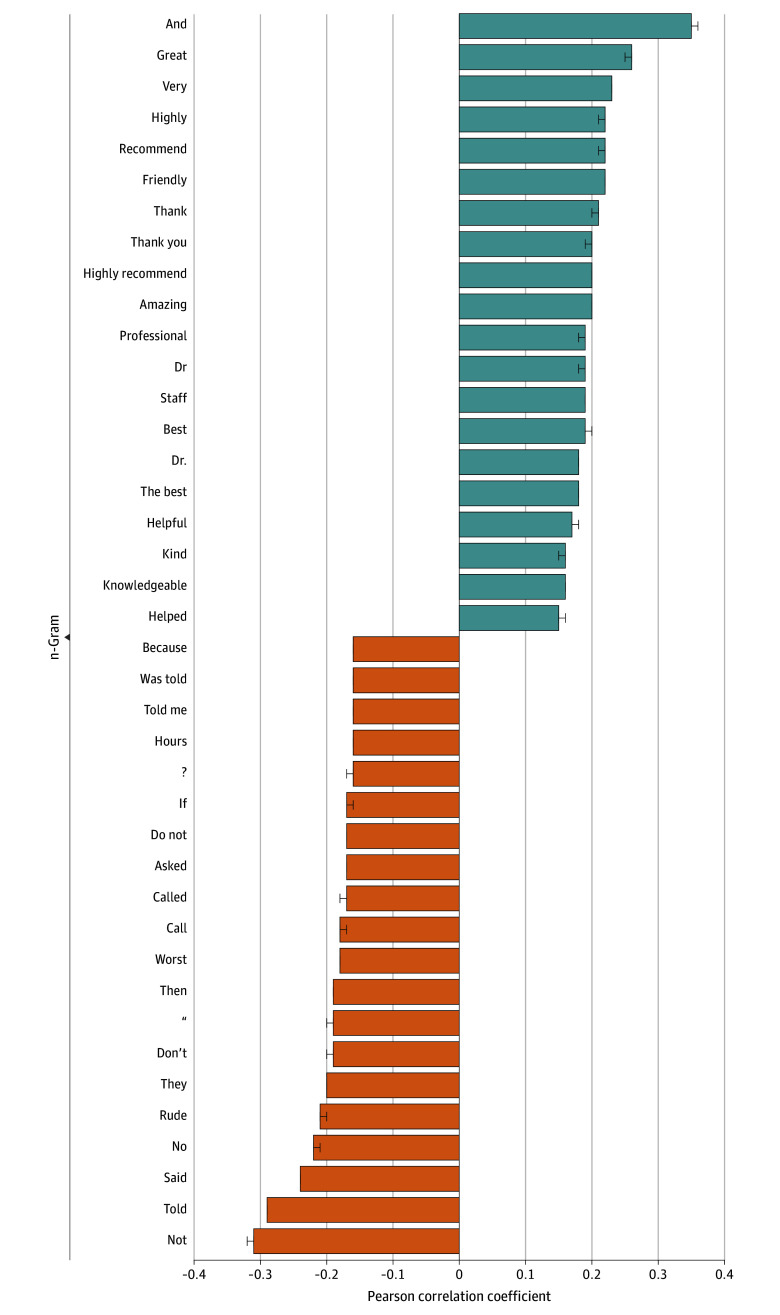
Bar Chart Displaying the Top 20 n-Grams Most Positively and Negatively Correlated With Positive (4-5 Star) Reviews Negative values indicate stronger correlations with negative (1-2 star) reviews. Error bars indicate 95% CIs.

Figure 1 displays n-grams most correlated with positive and negative reviews. Positive correlations represent larger correlations with positive reviews and negative correlations represent larger correlations with negative reviews. The word “not” was the most strongly correlated n-gram with negative reviews (*r* = 0.31; 95% CI, 0.31–0.32). For example, a 1-star review stated, “Despite calling numerous times, I was never given a quote so I had no idea what to expect. On arriving at the hospital on the day of the procedure, I was told that I could not check in since my insurance was not verified.”

In contrast, the word “and” was most correlated with positive reviews (*r* = 0.35; 95% CI, 0.35–0.36). For example, a 5-star review described, “I had a great experience during a procedure. Everyone was very attentive nice and helpful. I’m sorry for all the people who had bad experiences.”

The most correlated n-grams were generally consistent across health care facility type (eTable in Supplement 1). For example, “not” and “told” were among the top 3 n-grams most correlated with negative reviews across all facility types. Similarly, “and” was the most correlated n-gram with positive reviews for all but 3 facility types.

**Figure 2.**
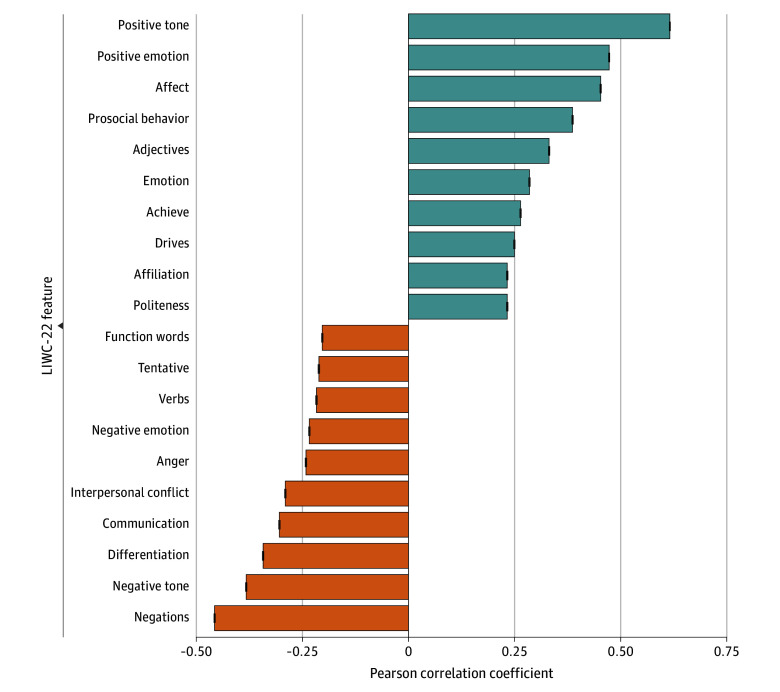
Bar Chart Displaying the Top 10 LIWC-22 Categories Most Positively and Negatively Correlated With Positive (4-5 Star) Reviews Negative values indicate stronger correlations with negative (1-2 star) reviews. LIWC-22 indicates 2022 Linguistic Inquiry and Word Count.

Figure 2 depicts LIWC-22 categories most correlated with positive and negative reviews. Negations (not, no, never, nothing) were most correlated with negative reviews (*r* = 0.46; 95% CI, 0.46–0.46), and positive tone (good, well, new, love) was most correlated with positive reviews (*r* = 0.62; 95% CI, 0.61–0.62). Latent Dirichlet allocation topic modeling identified topics associated with negative and positive reviews (Table 1 and Table 2). Topics correlated with negative reviews often emphasized poor communication and administrative challenges. For example, 1 topic correlated with negative reviews included terms such as “phone,” “hold,” and “call,” with a representative review stating, “They never answer the phone. It rings and rings until it hangs up on you. Good luck getting service.” Positive reviews were correlated with topics of staff friendliness, professionalism, and supportive care. One highly correlated topic included terms such as “recommend,” “highly,” and “professional,” exemplified by a review stating, “This place is wonderful! … [redacted] is fantastic–extremely knowledgeable, kind, helpful, and friendly.”

**Table 1.** Top 5 Topics Correlated With Negative Reviews Across All Facilities

Annotated theme or topic name	Top 15 words in topic	Illustrative Yelp post	Prevalence rank among 200 topics	Pearson correlation (95% CI)
Unfair payment	Money, waste, away, stay, scam, run, save, joke, real, complete, yourself, spend, lie, wasted, dollars	I paid in full and they still tried to go after my insurance for money. [Redacted] overly promises and under delivers. Waste of time and money. ZERO after care offered. STAY AWAY.	141	−0.251 (−0.253 to −0.249)
Poor treatment	Treat, horrible, sick, shut, joke, human, die, sad, god, shame, disgusting, lazy, deserve, crap, ashamed	I mean you receive treatment but its nothing fancy literally the bare minimum. nothing like [redacted] you have to beg for something if u want it.	95	−0.236 (−0.237 to −0.234)
Poor phone experience	Called, call, phone, asked, number, name, information, spoke, speak, hung, answered, calling, supervisor, woman, info	The phone number for this hospital is wrong if you tap the entry and hit call. The phone number is [redacted].	77	−0.230 (−0.232 to −0.229)
Phone waiting time	Phone, hold, call, minutes, answer, someone, put, calling, hung, answered, 20, speak, hang, phones, answers	They never answer the phone. It rings and rings until it hangs up on you. Good luck getting service.	98	−0.221 (−0.223 to −0.219)
Malpractice	Avoid, incompetent, costs, incompetence, serious, Sinai, death, negligence, risk, caused, dangerous, nightmare, mistake, malpractice, [redacted name of hospital system]	Questionable medical ethics. Very shocked that she is associated with [redacted]. Will be reporting this provider.	160	−0.210 (−0.211 to −0.208)

**Table 2.** Top 5 Topics Correlated With Positive Reviews Across All Facilities

Annotated theme	Top 15 words in topic	Example message	Prevalence rank among 200 topics	Pearson correlation (95% CI)
Kindness in care	Recommend, amazing, definitely, highly, love, absolutely, friends, such, wonderful, Melissa, Nicole, sweet, welcoming, 10/10	Had a great experience with nurse [redacted] she was very kind, knowledgeable and absolutely great service. She guide us thought the whole process and had absolutely great patience. Thank you nurse [redacted] you made our experience different.	138	0.323 (0.321-0.324)
Allaying anxiety	Kind, ease, calm, nervous, sweet, moment, gentle, cared, step, single, warm, anxiety, kindness, scared, remember	After an unfortunate kitchen accident Dr [redacted] quickly put me back together! It was a quick and easy experience despite a history with anxiety in medical situations. Dr [redacted] was amazing!	76	0.320 (0.319-0.322)
Professionalism	Recommend, highly, professional, anyone, friendly, knowledgeable, helpful, kind, caring, extremely, excellent, recommended, compassionate, attentive, thorough	This place is wonderful! [Redacted] is fantastic–extremely knowledgeable, kind, helpful, and friendly.	118	0.309 (0.308-0.311)
Helpful and easy	Super, friendly, nice, helpful, quick, easy, fast, clean, everyone, definitely, efficient, process, awesome, convenient, informative	Dr [redacted] is an excellent doctor, all nurses and CNA [redacted] are very nice helpful and friendly.	106	0.280 (0.279-0.282)
Over-expectations	Thank, beyond, above, Lisa, thanks, appreciate, grateful, kindness, special, chris, shout, appreciated, patience, professionalism, ladies	[Redacted] above and beyond w quick response and professionalism today. Super grateful for intelligence and helpfulness w urgent health issue thank you.	124	0.280 (0.278-0.281)

Abbreviation: CNA, certified nursing assistant.

## Discussion

This study utilizes the strength of natural-language processing to analyze a large volume of patient narratives from reviews on 1 online platform across US health care facilities, offering novel insights into evolving patient sentiment in the post-pandemic era. Key findings suggest that negative reviews frequently center on administrative inefficiencies (eg, insurance verification delays, poor communication) and linguistic markers of negation (eg, “not,” “told”), while positive reviews emphasize staff professionalism, kindness, and cohesive care experiences. Notably, the prominence of terms such as “and” in positive reviews, along with positive reviews being generally shorter than negative reviews, suggests that patients tend to briefly list multiple favorable aspects of their experiences. This study’s strengths include its large, diverse dataset spanning 2017 through 2023, advanced machine-learning techniques (eg, topic modeling, psycholinguistic analysis), and direct comparison of themes across 20 facility types, providing a comprehensive view of patient priorities.

Our findings align with prior work highlighting administrative hurdles and communication gaps as drivers of dissatisfaction,^18^ yet extend this literature by capturing shifts in patient sentiment post-pandemic. Unlike prior investigations limited to single settings or data collected before the pandemic, our analysis highlights “not” as an equally strong correlate with negative sentiment. This pattern is consistent with principles from expectation disconfirmation theory, which posits that dissatisfaction arises when a service experience falls short of prior expectations.^19^ In this context, expressions involving “not,” such as “not called,” “not informed,” or “not helpful,” may reflect perceived breakdowns in communication or process quality—areas where patients may have heightened expectations following the disruptions of the COVID-19 era. Collectively, these observations situate our work within a growing literature demonstrating how online platforms capture detailed facets of patient experiences potentially overlooked by standardized surveys.^7,10,11,12,18,20^ Recent work has also explored how unstructured review data can be systematically combined with structured hospital performance metrics to inform patient decision making and institutional comparisons.^21^ Such integrative approaches reinforce the value of leveraging both narrative and quantitative sources to evaluate health care quality more holistically.

For clinicians, administrators, and policymakers, these findings highlight specific service areas beyond clinical outcomes where interventions may be most impactful, such as administrative efficiency and empathetic communication. Health systems can use these insights to inform staff training, enhance service recovery protocols, and incorporate review-based indicators into patient experience dashboards to track emerging concerns in real time. For health care administrators, patterns of dissatisfaction related to insurance verification, scheduling delays, or unreturned calls point to actionable process improvements. Policymakers may find value in integrating online-review data into quality-measurement frameworks, using it as a complementary signal to capture issues such as transparency and access that may be underreported in standardized surveys. Finally, public health officials and researchers can leverage linguistic trends in reviews as part of sentiment surveillance or to prioritize qualitative domains in care equity initiatives.

While this study highlights how administrative hurdles and communication breakdowns frequently drive negative online reviews, it is essential to interpret these findings in the broader context of health care quality. Patient satisfaction is an important domain of care, but it does not fully capture clinical effectiveness. Communication inefficiencies—such as difficulty reaching clinicians or facilities or confusion around billing—may erode trust and engagement, but they are ultimately only meaningful insofar as they affect patients’ access to, and understanding of, high-quality clinical care.

Improvements in communication should therefore be seen not as standalone goals but as enablers of clinical excellence. When patients are informed and respected, they can be more likely to follow treatment plans and report positive outcomes.^22^ Conversely, administrative inefficiencies may serve as early warning signals of deeper structural issues that could indirectly compromise clinical outcomes. Future research should explore these interdependencies more directly, examining how experiences captured in online reviews relate to care quality metrics such as diagnostic accuracy, treatment adherence, or hospital readmissions.

### Limitations

This study has several limitations. First, users of this online platform may skew toward younger, tech-savvy populations, limiting generalizability. Second, the platform’s focus on public reviews risks overrepresentation of extreme sentiments (positive or negative), particularly during crises such as the COVID-19 pandemic. Third, while this online platform applies proprietary filtering to suppress fake or low-trust content, we did not independently validate the authenticity of reviews, and some reviews in our dataset may be inauthentic. Fourth, categorizing facilities by “essential health benefits” excluded niche services, potentially omitting unique patient concerns. Moreover, our analysis did not examine how patient sentiment differed by facility characteristics such as size, public or private ownership, or patient volume. Future research should investigate whether such characteristics are associated with systematic differences in review sentiment, potentially uncovering structural drivers of patient experience. Additionally, while our data span the COVID-19 pandemic, we did not perform temporal stratification on the pandemic period. Patient reviews during this time may reflect experiences uniquely influenced by pandemic-related disruptions, such as limited in-person access, visitation restrictions, or heightened emotional stress. Future work should explicitly examine temporal trends to isolate the influence of COVID-19 on patient sentiment.

## Conclusions 

This analysis highlights common communication challenges and operational issues frequently mentioned in online patient narratives, offering a window into how patients perceive care quality. Our findings reveal correlations between certain themes and review ratings, and recognizing these recurring concerns may help inform areas for further study and support the design of patient-centered initiatives. Future research should integrate multiplatform data and explore how these themes relate to clinical outcomes to better understand their potential implications for equitable, patient-centered care improvement.

## Notes

Supplement 1. **eMethods.** Yelp Facility Types Included in Analysis **eTable.** Top 5 n-Grams Correlated With Positive and Negative Reviews by Facility Type

Supplement 2.
Data Sharing Statement
