# Supplementary material for: Online Reviews of Health Care Facilities
Source: JAMA Netw Open. 2025 Aug 1;8(8):e2524505. doi: 10.1001/jamanetworkopen.2025.24505 (PMC12317347; doi:10.1001/jamanetworkopen.2025.24505)
Supplement: Supplement 1. — eMethods. Yelp Facility Types Included in Analysis eTable. Top 5 n-Grams Correlated With Positive and Negative Reviews by Facility Type [file jamanetwopen-e2524505-s001.pdf]

Supplemental Online Content

Sehgal NKR, Guntuku SC, Southwick L, Merchant RM, Agarwal AK. Online reviews of health care facilities. *JAMA Netw Open.* 2025;8(8):e2524505. doi:10.1001/jamanetworkopen.2025.24505

eMethods. Yelp Facility Types Included in Analysis

eTable. Top 5 n-Grams Correlated With Positive and Negative Reviews by Facility Type

This supplemental material has been provided by the authors to give readers additional information about their work.

© 2025 Sehgal NKR et al. *JAMA Network Open*.

eMethods. Yelp facility types included in analysis

The facilities included were urgent care clinics, medical centers, physical therapy, pharmacies, physicians, hospitals, counseling and mental health, rehabilitation centers, emergency departments, occupational therapy, home health care, skilled nursing, ultrasonography imaging centers, lactation services, prenatal care, speech therapists, diagnostic services, reproductive health centers, nurse practitioners, and dialysis clinics.

© 2025 Sehgal NKR et al. *JAMA Network Open*.

eTable . Top 5 n-grams correlated with positive and negative reviews by facility type. N-grams are ordered from highest to lowest correlation.

Facility Type	Number of Reviews	Mean Star Rating	Negative Reviews	Positive Reviews
Urgent Care	233266	3.0	'told', 'not', 'said', "", 'rude'	'and', 'very', 'staff', 'friendly', 'great'
Medical Centers	212039	2.69	'not', 'told', 'said', 'no', ""	'and', 'great', 'very', 'staff', 'friendly'
Physical Therapy	176888	4.07	'not', 'told', 'said', 'called', 'no'	'and', 'highly', 'recommend', 'great', 'highly recommend'
Pharmacy	155115	2.55	'not', 'told', 'rude', 'said', 'worst'	'great', 'friendly', 'helpful', 'and', 'best'
Hospitals	130603	2.44	‘not', 'told', 'no', 'said', 'hours'	'and', 'thank', 'great', 'thank you', 'staff'
Physicians	75097	2.73	'not', 'told', 'said', 'rude', 'they'	'dr', 'and', 'dr .', 'great', 'very'
Counseling & Mental Health	76652	3.34	‘not', 'they', 'told', 'no', 'said'	'and', 'life', 'highly', 'helped', 'recommend'
Rehabilitation Center	52306	3.15	'not', 'told', 'no', 'do not', 'they'	'and', 'great', 'highly', 'amazing', 'recommend'
Occupational Therapy	37713	3.46	'not', 'told', 'no', "don't", 'said'	'and', 'great', 'highly', 'recommend', 'therapy'
Emergency Rooms	37205	2.79	‘told', 'not', 'said', "", 'hours']	'and', 'staff', 'very', 'great', 'thank'
Home Health Care	35961	3.20	'not', 'told', 'no', 'said', 'call'	'and', 'care', 'highly', 'great', 'recommend'
Skilled Nursing	26859	2.92	'not', 'no', 'told', 'do not', "don't"	'and', 'staff', 'great', 'very', 'thank'
Ultrasound Imaging Centers	24479	3.98	'not', 'told', 'said', 'call', 'rude'	'and', 'experience', 'great', 'baby', 'recommend'
Prenatal	16158	4.29	'not', 'told', 'said', 'appointment', 'they'	'and', 'amazing', 'recommend', 'her', 'highly'
Lactation Services	15980	4.65	'not', 'said', 'told', 'rude', 'they'	'and', '. she', 'our', 'her', 'she'
Speech Therapists	15024	3.90	'not', 'told', 'no', 'call', "don't"	'and', 'speech', 'with', 'has', 'son'
Diagnostic Services	12962	3.09	'not', 'told', 'said', 'call', 'rude'	'and', 'very', 'great', 'friendly', 'staff'
Reproductive Health Services	11492	3.83	'told', 'not', 'said', 'rude', ""	'and', 'dr', 'dr .', 'very', 'staff'
Nurse Practitioner	6044	3.89	'not', 'told', 'said', 'they', 'called'	'and', '!', 'is', 'highly', 'amazing'
Dialysis Clinics	1252	2.91	'not', 'told', 'no', "don't", 'rude'	'friendly', 'and', 'great', 'very', 'staff'

© 2025 Sehgal NKR et al. *JAMA Network Open*.
