# Supplementary material for: Online Reviews of Health Care Facilities
Source: JAMA Netw Open. 2025 Aug 1;8(8):e2524505. doi: 10.1001/jamanetworkopen.2025.24505 (PMC12317347; doi:10.1001/jamanetworkopen.2025.24505)
Supplement: Supplement 2. — Data Sharing Statement [file jamanetwopen-e2524505-s002.pdf]

Data Sharing Statement

Sehgal. Online Reviews of Health Care Facilities. *JAMA Netw Open*. Published August 01, 2025. doi:10.1001/jamanetworkopen.2025.24505

Data
Data available: No
